# Revealing the impact of local access-site complications and upper extremity dysfunction post transradial percutaneous coronary procedures

**DOI:** 10.1007/s12471-015-0747-9

**Published:** 2015-10-05

**Authors:** E.M. Zwaan, A.G.M.M. Koopman, C.A.J. Holtzer, F. Zijlstra, M.J.P.F. Ritt, G. Amoroso, E. Moerman, M.J.M. Kofflard, A.A.J. IJsselmuiden

**Affiliations:** 1 Department of Cardiology, Albert Schweitzer Hospital, Dordrecht, the Netherlands; 2 Department of Plastic, Reconstructive and Hand Surgery, Albert Schweitzer Hospital, Dordrecht, the Netherlands; 3 Department of Cardiology, Erasmus University, Rotterdam, the Netherlands; 4 Department of Plastic, Reconstructive and Hand Surgery, VU University, Amsterdam, the Netherlands; 5 Department of Cardiology, Onze Lieve Vrouwe Gasthuis, Amsterdam, the Netherlands; 6 Department of Plastic, Reconstructive and Hand Surgery, Onze Lieve Vrouwe Gasthuis, Amsterdam, the Netherlands

**Keywords:** Radial artery, Access-site complication, Upper extremity dysfunction

## Abstract

**Objectives:**

Little is known about local access-site complications and upper extremity dysfunction after transradial percutaneous coronary procedures (TR-PCP). This systematic review study aimed to summarise the current knowledge on the incidences of access-site complications and upper extremity dysfunction after TR-PCP.

**Methods:**

Two independent, trained investigators searched MEDLINE, EMBASE and CENTRAL for eligible studies published before 1 January 2015. Also, they hand-searched the conference proceedings of the annual scientific sessions of the American College of Cardiology, the American Heart Association, European Society of Cardiology, and the Trans-catheter Cardiovascular Therapeutics. Inclusion criteria were cohort studies and clinical trials discussing the incidence of access-site complications and upper extremity function after transradial percutaneous coronary intervention (TR-PCI) and/or transradial coronary angiography (TR-CAG) as endpoints.

**Results:**

176 articles described access-site complications. The incidence is up to 9.6 %. Fourteen articles described upper extremity dysfunction, with an incidence of up to 1.7 %. Upper extremity dysfunction was rarely investigated, hardly ever as primary endpoint, and if investigated not thoroughly enough.

**Conclusion:**

Upper extremity dysfunction in TR-PCP has never been properly investigated and is therefore underestimated. Further studies are needed to investigate the magnitude, prevention and best treatment of upper extremity dysfunction. Optimising TR-PCP might be achieved by using slender techniques, detection of upper extremity dysfunction and early referral to a hand rehabilitation centre.

**Electronic supplementary material:**

The online version of this article (doi: 10.1007/s12471-015-0747-9) contains supplementary material, which is available to authorized users. This supplementary file contains References 51–202.

## Introduction

**Fig. 1:**
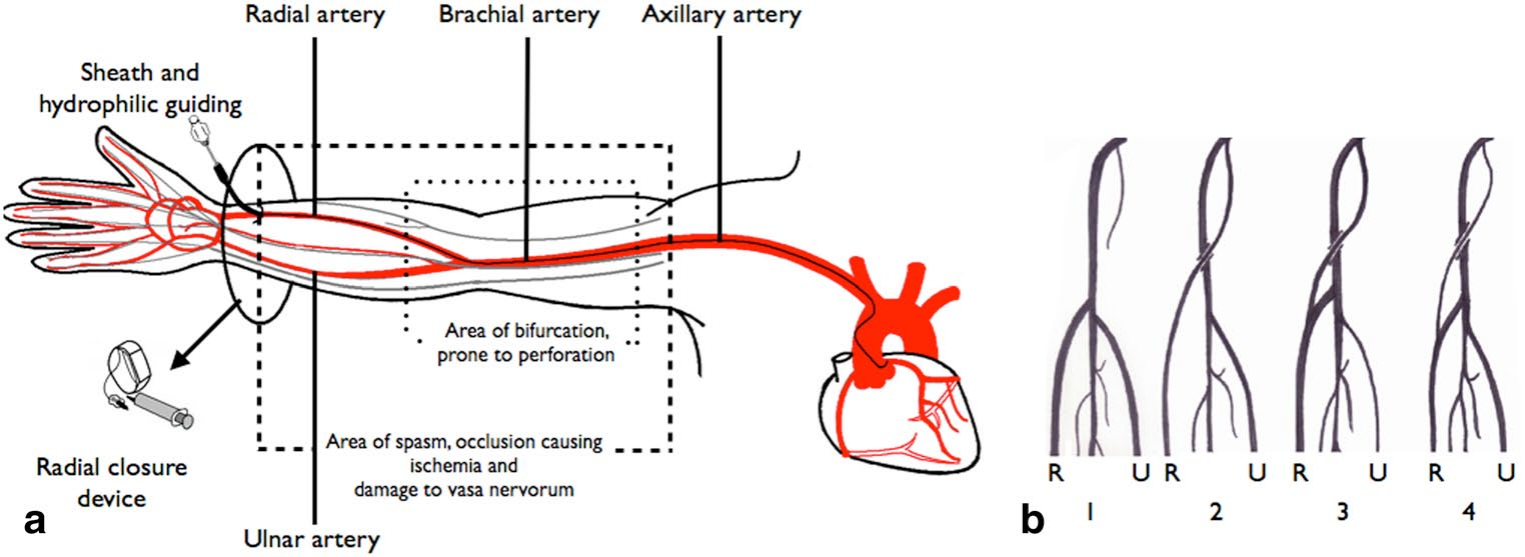
The anatomy of the upper extremity (**a**) and its variations (**b**). **a** The anatomy of the arteries (*red line*) and nerves (*grey line*) of the arm leading to the heart. The area where the bifurcation of the radial artery might occur is accentuated; this area is prone to perforation (*inner dashed box*). The area where spasm, occlusion or damage to vasa nervorum occurs is also highlighted (*outer dashed box*). Hydrophilic guiding catheters and special radial access closure devices might reduce the incidence of these complications and could diminish the impact on upper extremity function. **b** Frequent variations of the take-off of the radial artery. The radial artery ® and ulnar artery (U) are illustrated. *1.* Radial artery arising from the brachial artery. *2.* Independent radial artery arising from the axillary artery. *3.* Radial artery arising from the axillary artery with a contribution from the brachial artery. *4.* Slender artery arising from the axillary artery continuing as the radial artery. The major blood supply to the radial artery is supplied by the brachial artery. This type is highly susceptible to perforation.

**Fig. 2:**
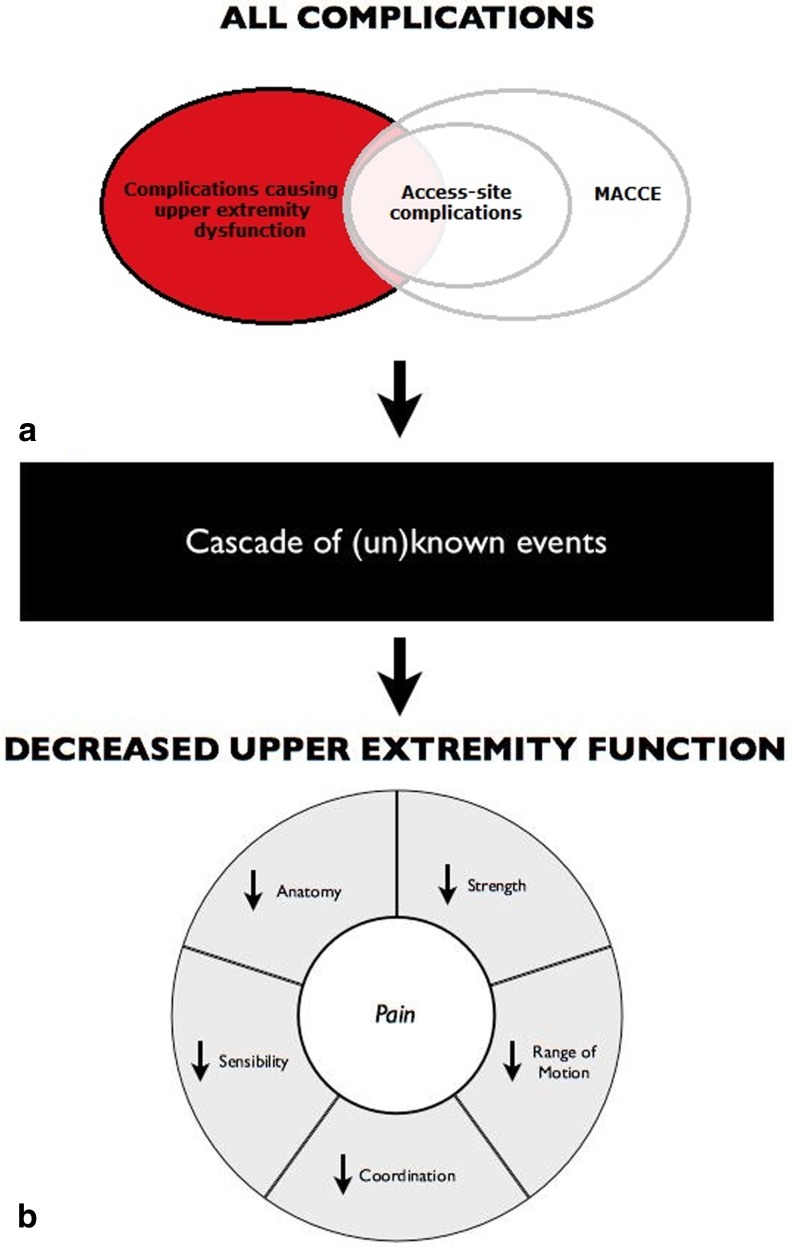
Complications after transradial percutaneous coronary procedures (TR-PCP) and the impact on upper extremity function. All complications affect upper extremity function after TR-PCP. The mechanism and the magnitude in which they affect function is partly known and partly unknown (*black box*). The circle below represents upper extremity function and the negative impact complications might have. **a** Very little is known about complications affecting upper extremity function after TR-PCP (*red circle* without overlap). However, there is awareness for the overlap part. Access-site complications (*inner white circle*) are described in the literature (Table 1). Major adverse cardiac and cerebral events (MACCE) (*outer white circle*) might influence upper extremity function as well, but were not investigated in this article. **b** Upper extremity function consists of several important physiological capacities as mentioned in the pie diagram. Pain is not a physiological parameter, but negatively affects all parameters of upper extremity function

Little is known about the impact of access-site and procedural complications on upper extremity function after transradial percutaneous coronary interventions (TR-PCI) and transradial coronary angiography (TR-CAG) even though the transradial route is quickly becoming the golden standard for many interventional cardiologists [1]. In 2013, over 85 % of all PCIs at our hospital were performed using the radial artery. In comparison, in the third quarter of 2012 only 16.9 % of all PCIs in the USA were performed using this approach [2]. This appreciation of TR-PCI and TR-CAG, summed up under the heading of the transradial percutaneous coronary procedures (TR-PCP), stems from innovations in the field of material science. Refinement of materials, such as hydrophilic sheaths and miniaturisation of equipment, has increased the therapeutic options, thus making TR-PCP elegant, safe and feasible [2–4]. This was confirmed in a comprehensive meta-analysis of randomised clinical trials by Jolly et al. [3]. They compared radial versus femoral access and showed a 73 % reduction in major bleeding and a trend towards reductions of mortality, myocardial infarction and stroke in favour of the radial route [3]. Additionally, TR-PCP is associated with lower costs and higher patient satisfaction, correlating with a higher quality of life [4]. However, TR-PCP is technically more challenging with a long learning curve [5–7], partly due to the complex anatomical variability of the nerves and blood vessels in the upper extremity (Fig. 1; [8–10]) This makes TR-PCP more susceptible to functional complications. To understand the impact of access-site complications and its effect on upper extremity function a clear definition of the latter is needed and this has been much debated. Traditional clinical assessment has focused on grip or pinch strength and range of motion [11, 12]. A much more encompassing definition would be ‘the physiological capacity in which a patient can use an anatomically unaffected upper limb in everyday activities’. To evaluate this, several aspects should be considered. It comprises anatomy, including blood and lymph circulation, muscle strength, active range of motion, coordination and sensory functions. Pain affects all of these parameters (Fig. 2). Adequate knowledge of complications is necessary to prevent dysfunction. Current reviews have not described upper extremity function [13–15]. This study aimed to summarise the current knowledge on the incidences of access-site complications and upper extremity dysfunction after TR-PCP.

**Table 1:** Pooled incidence of complications. Pooled incidence in percentages and range in brackets of reported access-site complications after TR-PCP, TR-PCI and TR-CAG

Complication	Mean incidence after TR-PCP (%)	Mean incidence after TR-PCI (%)	Mean incidence after TR-CAG (%)	Mean incidence after pooled procedures (%)	References
Upper extremity dysfunction	0.32 % (0.07–1.51)	0.81 % (0.16–3.91)	0.12 % (0.02–0.72)	0.32 % (0.10–1.01)	[20–33]
Upper extremity ischaemia	0.14 % (0.04–0.57)	0.29 % (0.16–0.53)	0.19 % (0.04–0.99)	0.21 % (0.10–0.43)	[8, 21, 22, 24, 29–31, 33–66]
Pain	8.03 % (3.97–15.55)	4.49 % (0.64–25.43)	9.57 % (7.52–12.11)	7.65 % (4.51–12.67)	[25, 28, 34, 35, 49, 67–75]
Radial artery spasm	8.7 % (5.8–12.7)	4.24 % (2.47–7.17)	7.25 % (4.01–12.77)	0.5 % (0.01–2.75)	[26, 30, 31, 34, 35, 55, 56, 67, 71, 73, 76–98]
Severe radial artery spasm	1.82 % (1.07–3.06)	1.13 % (0.68–1.87)	1.58 % (0.85–2.93)	1.45 % (1.07–1.96)	[3, 8, 22, 24, 34, 35, 37, 44, 46, 53, 55, 65, 66, 71, 72, 76, 78, 79, 81–85, 89, 93, 94, 95, 99–113]
Early radial artery occlusion	4.0 % (2.67–5.94)	2.59 % (1.81–3.69)	4.98 % (2.34–10.31)	3.45 % (2.59–4.58)	[8, 21, 22, 24, 26, 27, 29–31, 33, 35, 38, 43–47, 49, 50, 52, 54, 55, 57, 59, 61, 62, 69, 70, 72, 74, 79, 96, 97, 100, 101, 104, 115–140]
Late radial artery occlusion	3.23 % (2.01–5.15)	3.21 % (2.10–4.87)	3.30 % (1.98–5.46)	3.34 % (2.57–4.32)	[20, 21, 23–27, 31, 36, 44, 46, 48, 53, 55–57, 61, 65, 67, 71, 72, 76, 86, 89, 93, 103–105, 115, 125, 130, 133, 136, 140–143]
Minor access-site bleeding	4.30 % (2.35–7.75)	1.93 % (0.61–5.96)	1.56 % (0.93–2.61)	2.49 % (1.29–4.75)	[6, 24, 26, 29, 34, 36, 47–49, 55, 59, 64, 76, 78, 80, 107, 108, 116, 119, 121, 127, 131, 132, 136–138, 144–150]
Major access-site bleeding	0.34 % (0.12–0.97)	0.79 % (0.50–1.23)	0.22 % (0.03–1.57)	0.66 % (0.44–0.99)	[25, 36, 40, 43, 45, 46, 56, 60–62, 64, 65, 74, 79, 101–103, 106, 112, 118, 123, 130, 136–138, 144, 147, 148, 151–166]
Minor access-site haematoma	3.89 % (2.40–6.25)	3.34 % (2.49–4.47)	1.54 % (0.54–4.32)	3.22 (2.42–4.28)	[8, 23, 26, 27, 29–31, 33, 36, 37, 39, 44, 45, 47, 57, 58, 68, 71, 72, 76, 80, 86, 89, 92, 95–97, 100, 103, 107, 108, 115, 116, 118, 119, 122, 124, 130, 132, 138, 146, 152, 157, 167–175]
Major access-site haematoma	0.87 % (0.56–1.36)	1.07 % (0.67–1.71)	0.45 % (0.24–0.82)	0.89 % (0.65–1.21)	[6, 8, 23, 25, 27, 30, 33, 34, 37, 39–41, 43, 45, 49–55, 68–70, 72–74, 76, 80, 86, 9 0, 100, 101, 105, 107, 108, 110–113, 116–119, 123–125, 127, 129, 130, 132, 134, 136, 139, 142, 145, 146, 150, 152, 153, 155–159, 162, 163, 165, 168, 169, 172, 174–189]
Perforation	0.28 % (0.08–0.90)	0.64 % (0.12–3.22)	0.48 (0.16–1.49)	0.40 % (0.20–0.80)	[37, 47, 65, 66, 67, 90, 93, 95, 113, 136, 150, 177, 184, 190, 191]
Dissection	0.40 % (0.10–1.59)	0.48 % (0.09–2.39)	0.72 % (0.15–3.43)	0.49 % (0.19–1.27)	[8, 20, 22, 24, 26, 27, 37, 41, 50, 51, 73, 90, 101, 102, 186, 190, 192]
Swelling	2.4 % (1.1–5.3)	3.5 % (0.5–20.6)	1.0 (0.1–6.6)	2.76 % (1.36–5.5)	[28, 34, 35, 80, 154]
Compartment syndrome	0.01 % (0.0–0.02)	0.14 % (0.03–0.71)	0.0 % (0.0–0.11)	0.0 % (0.01–0.07)	[8, 32, 37, 45, 51, 56, 173, 184]
Pseudo-aneurysm	038 % (0.20–0.73)	0.32 % (0.18–0.58)	0.18 % (0.05–0.73)	0.32 % (0.21–0.49)	[20, 22, 24, 25, 27, 30, 31, 36–38, 40, 41, 43–45, 50, 51, 55–57, 60–62, 69, 73, 96, 103, 112, 122, 124, 137–139, 144–146, 148, 155, 157, 166, 171–173, 180, 181, 183, 184, 188, 192]
Arteriovenous fistula	0.20 % (0.09–0.48)	0.22 % (0.11–0.43)	0.19 % (0.05–0.74)	0.21 % (0.13–0.35)	[25–27, 30, 31, 36–38, 40, 41, 43–45, 50, 51, 56, 57, 60–62, 95, 112, 113, 117, 136–138, 142, 144, 155, 166, 179, 184, 188, 191]
Infection/Inflammation	0.83 % (0.34–1.99)	1.06 % (0.15–7.17)	Not applicable	0.86 % (0.38–1.93)	[22, 28, 37, 73, 137, 171, 193]

*TR-CAG* transradial catheterisation, *TR-PCI* transradial percutaneous coronary intervention, *TR-PCP* transradial percutaneous coronary procedures.

## Methods

Two independent, trained investigators searched MEDLINE, EMBASE and CENTRAL for eligible studies published before 1 January 2015. Search keywords included: “radial artery”, “percutaneous coronary intervention”, “coronary angiography” and “complications”. Various combinations of these terms were used depending on the requirements of the database. Language was not restricted. The investigators hand-searched the conference proceedings of the annual scientific sessions of the American College of Cardiology, the American Heart Association, European Society of Cardiology, and the Trans-catheter Cardiovascular Therapeutics. Major reviews regarding the radial approach for coronary procedures were systematically searched. Cross-references and quoted papers were checked, and experts were contacted to identify other relevant trials.

### Selection criteria

Inclusion criteria were cohort studies and clinical trials discussing the incidence of access-site complications and upper extremity function after TR-PCI and TR-CAG as endpoints. Due to the limited information available, we used some case reports for a subsection regarding upper extremity dysfunction. Editorials, reviews and letters were excluded, as well as articles with incomplete data.

### Endpoint definitions

The primary clinical outcome was upper extremity dysfunction, defined as loss of strength, sensory loss, coordination loss and/or loss of active range of motion, ascertained by patient history and/or through physical examination. Upper extremity ischaemia was defined as necrosis, symptomatic embolisation or thrombosis and claudication. Pain was defined as paraesthesia and/or a visual analogue score of ≥ 5 per procedure, post procedure or at follow-up. Non-severe radial artery spasm was defined as operator perceived or radiological confirmed spasm, while severe radial artery spasm was the inability to advance the guide wire and/or the inability to remove the sheath. Furthermore, radial artery occlusion was defined as a completely occluded artery confirmed by Doppler or angiography during hospital admission (early) or at follow-up (late). Access-site haematoma was defined as minor (< 5 cm) or major (≥ 5 cm); access-site (prolonged) bleeding as requiring additional compression (minor) or blood transfusion and/or a haemoglobin drop of ≥ 3 mmol/L (major). Perforation was confirmed by angiography and dissection by angiography. Swelling and oedema were assessed visually and not attributed to bleeding. Compartment syndrome, as diagnosed by the operator, and requiring treatment; pseudo-aneurysm, requiring treatment; arteriovenous fistula, as diagnosed by the operator; and inflammation was defined as local inflammation, abscess and/or mycotic aneurysms.

## Data analysis and synthesis

Statistical analysis was performed using the R software programming language [16]. Pooled estimates were calculated for the incidence of previously mentioned clinical outcomes. A summary of pooled-effect estimates and corresponding 95 % CIs was derived by using the DerSimonian-Laird random-effects model [17]. The random-effects method was chosen because it incorporates between- and within-study variance. To assess heterogeneity a Cochrane Q-statistic test and the I^2^ statistic were used [18]. Funnel plots were used to assess publication bias [19]. If high heterogeneity was observed, meta-regression analysis, using the test of moderators, was conducted.

## Results

**Fig. 3:**
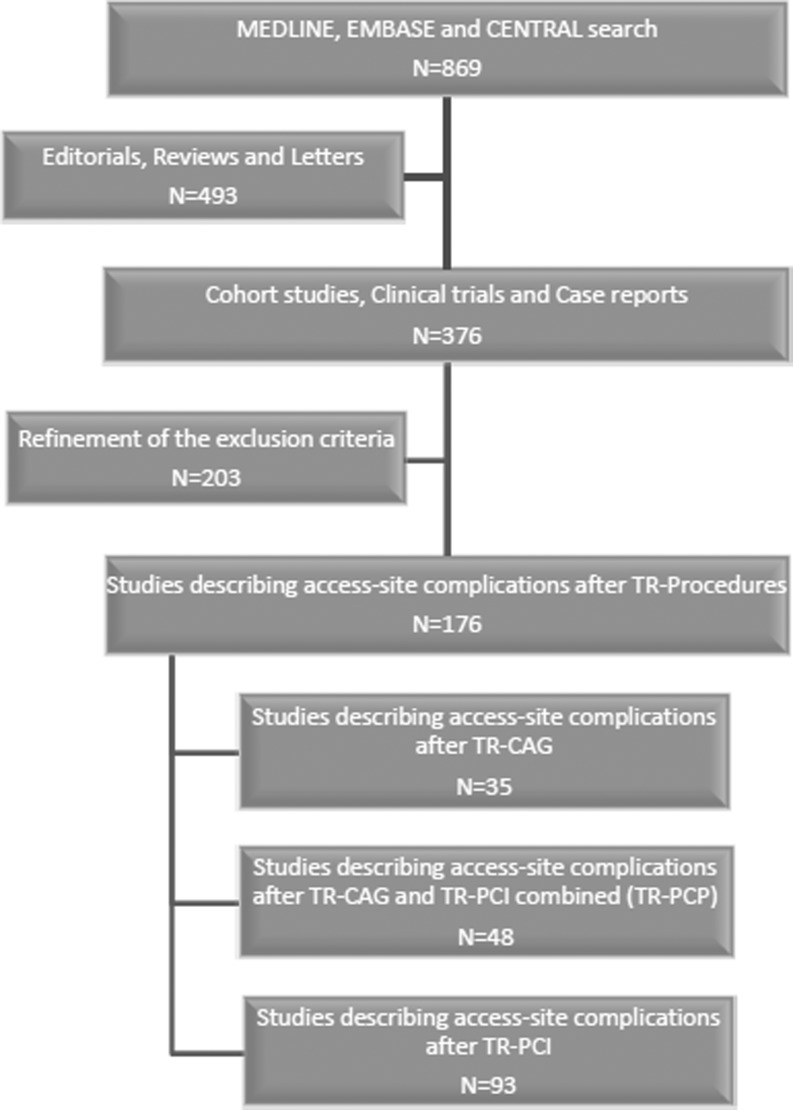
Inclusion flow diagram. Using MEDLINE, EMBASE and CENTRAL 869 articles were obtained concerning access-site complications after trans radial percutaneous coronary procedures (TR-PCP). There were 376 cohort studies and clinical trials. After careful selection 176 articles were included based on the inclusion criteria as described in the methods

A total of 869 citations were evaluated. After careful selection 176 papers were eligible (Fig. 3). The pooled incidence rates of upper extremity complications after TR-PCP, TR-PCI and TR-CAG are shown in Table 1. We did produce funnel and forest plots, but because of the extent of all the results, we do not show these in this article.

### Upper extremity dysfunction

Fourteen articles described upper extremity dysfunction after TR-PCP, with a pooled incidence of up to 1.7 % (Table 1). Two articles objectively measured upper extremity dysfunction after TR-PCP. Wu et al. described hand-strength of both arms retrospectively 1 year after TR-PCI. Twenty-four patients underwent 8Fr coronary procedures and 16 patients 6Fr procedures. There was no significant difference between groups, nor when comparing opposite arms [20]. Valgimigli et al. showed that Allen’s test did not influence handgrip strength, as was measured before, at 1 day, 1 month and 1 year after the procedure [21]. Evaluation of functional loss by history of follow-up was reported in three articles [22–24]. Prull et al. described 93 patients who underwent TR-PCP. None of them reported sensory loss, diminished strength, coordination and/or active range of motion at 10-day follow-up [22]. Likewise, no functional complaints were reported by De Belder et al. They described 75 patients who underwent TR-PCP with a follow-up at 4–6 weeks. Nevertheless, they found 1 patient with transient paraesthesia [23]. While Kiemeneij et al. described 100 cases of radial artery occlusion after TR-PCI, no cases of upper extremity dysfunction were found at 1–3 months of follow-up. Functional disability was defined as the ability to repeatedly open and close the catheterised hand 50 times [24]. Nerve damage was mentioned as an endpoint in two articles. They found no cases of nerve damage [25, 26]. Seven articles, where functional outcome was not a solitary endpoint, described cases with nerve damage [27–33]. Lotan et al. described a case with sensory loss of the thumb and index finger at 1 month [27]. Similarly, Tharmaratnam et al. reported 22 patients with either distal (typically thumb) pain, or paraesthesia in the hand [28]. Additionally, Chatelain et al. described 159 patients who underwent TR-PCP; one of these patients with chronic radial occlusion complained of paraesthesia in the right thumb during exercise at discharge [29]. Tewari et al. found three patients who developed transient neurological deficit post procedure, without any residual deficit [30]. Median nerve compression due to a large haematoma, which resolved within 48 h, was reported by Spaulding et al. [31]. In another case described by Tizon-Marcos et al. a pseudo-aneurysm resulted in compartment syndrome causing a Volkmann’s contracture, a debilitating permanent claw-like deformity [32]. Zankl et al. reported a case of persistent radial thrombosis, with unknown casualty, leading to radial nerve paresis [33].

### Upper extremity ischaemia

A total of 45 articles described upper extremity dysfunction after TR-PCP, with a pooled incidence of up to 0.3 % (Table 1; [8, 21, 22, 24, 29–31, 33–66]) The majority of articles described no ischaemia or claudication. However, necrosis leading to amputation of the index finger was described in 1 article [34]. Timely intervention with appropriate methods may reduce the need for amputations.

## Pain

Severe pain (VAS ≥ 5) was reported in 16 articles and had a pooled incidence of up to 9.6 % (Table 1; [25, 28, 34, 35, 49, 67–75]) The majority of the articles described peri-procedural pain. Studies used a wide variety of scoring methods, whereupon we excluded many studies. Since there was no unequivocal classification, findings must be interpreted carefully. Pain, especially when chronic, affects all functional parameters and can be very debilitating. Knowledge of causality and location is essential for treatment and prevention; neither were described. Functional outcome was not described either.

### Radial artery spasm

Analysis of 36 articles showed a pooled incidence of up to 8.7 % for non-severe radial artery spasm (Table 1; [26, 30, 31, 34, 35, 55, 56, 67, 71, 73, 76–98]).

Severe radial artery spasm showed a pooled incidence of up to 1.8 % extracted from 46 articles (Table 1; [3, 8, 22, 24, 34, 35, 37, 44, 46, 53, 55, 65, 66, 71, 72, 76, 78, 79, 81–85, 89, 93–95, 99–113]) There is a wide variety in procedural and evaluation methods, with non-severe radial artery spasm reported at an incidence of up to 50 % [75], and severe radial artery spasm at up to 15 % [77]. There is a significant relationship between radial spasm and the baseline artery diameter, type of spasmolytic drug, the amount of catheter changes and the administered volume of contrast medium. Another independent predictor of radial artery spasm is female gender [71, 78, 81, 83, 85, 97, 114]. Data concerning timing, duration and location of arterial spasm were sparse [34, 75, 80, 84, 85]. One article described subsequent ischaemia [34]. However, none of the articles reported functional outcome.

### Radial artery occlusion

Sixty-six articles mentioned early radial artery occlusion, with pooled incidence rates of up to 5.0 % (Table 1; [8, 21, 22, 24, 26, 27, 29–31, 33, 35, 38, 43–47, 49, 50, 52, 54, 55, 57, 59, 61, 62, 69, 70, 72, 74, 79, 96, 97, 100, 101, 104, 115–140]). Late radial artery occlusion was described in 43 articles and had an incidence of up to 3.3 % (Table 1; [20, 21, 23–27, 31, 36, 44, 46, 48, 53, 55–57, 61, 65, 67, 71, 72, 76, 86, 89, 93, 103–105, 115, 125, 130, 133, 136, 140–143]). Radial artery occlusion is often asymptomatic or subclinical (e.g. only complaints during cold weather or heavy exertion). This lack of clinical consequences is attributed to the dual blood supply of the hand and the formation of collateral blood vessels. Ischaemia and subsequent necrosis due to radial artery occlusion have been reported [34, 37, 50]. In the current reviewed studies, a broad variety of diagnostic methods was used. The majority of the studies diagnosed occlusion with Doppler echography, and only these protocols were included. Spontaneous recanalisation, without prolonged treatment, took place in the majority at 30 days to 3 months post-procedure [26, 33, 44, 55, 61, 96, 126, 127]. Occlusion of arteries of the upper extremity other than the radial artery is not reported. However, location of the occlusion was often not investigated by angiography or echography. The majority of the articles did not investigate functional outcome.

### Access-site bleeding

Minor access-site bleeding was reported in 34 articles, with a pooled incidence of up to 4.3 % (Table 1; [6, 24, 26, 29, 34, 36, 47–49, 55, 59, 64, 76, 78, 80, 107, 108, 116, 119, 121, 127, 131, 132, 136–138, 144–150]). Forty-five articles reported on major access-site bleeding at a pooled rate up to 0.8 % (Table 1; [25, 36, 40, 43, 45, 46, 56, 60–62, 64, 65, 74, 79, 101–103, 106, 112, 118, 123, 130, 136–138, 144, 147, 148, 151–166]). Both major and minor access-site bleeding are often described in the literature. Even though bleeding and subsequent swelling might have a debilitating impact on functional outcome none described the impact of bleeding on functional outcome.

### Access-site haematoma

Fifty-five articles reported on minor access-site haematoma, with a pooled incidence of 3.9 % (Table 1; [8, 23, 26, 27, 29–31, 33, 36, 37, 39, 44, 45, 47, 57, 58, 68, 71, 72, 76, 80, 86, 89, 92, 95–97, 100, 103, 107, 108, 115, 116, 118, 119, 122, 124, 130, 132, 138, 146, 152, 157, 167–175]). Major access-site haematoma was reported with a pooled rate of up to 1.1 % found in 88 articles (Table 1; [6, 8, 23, 25, 27, 30, 33, 34, 37, 39–41, 43, 45, 49–55, 68–70, 72–74, 76, 80, 86, 90, 100, 101, 105, 107, 108, 110–113, 116–119, 123–125, 127, 129, 130, 132, 134, 136, 139, 142, 145, 146, 150, 152, 153, 155–159, 162, 163, 165, 168, 169, 172, 174–189]). Haematomas ranged from ecchymosis to haematomas requiring surgery. Compression of the median nerve was described as a result of a major haematoma [31].Haematoma and subsequent compression of nerves, blood vessels and surrounding tissue might have a debilitating impact. Yet, the majority of the articles did not describe location and functional outcome in relationship to haematoma.

### Perforation

Pooled incidence of perforation was reported as up to 0.5 % in 14 articles (Table 1; [37, 47, 65–67, 90, 93, 95, 113, Pooled incidence of perforation was reported as up to36, 150, 177, 184, 190, 191]). Perforation of the radial artery or its adjacent vessels by the guide wire can result in several complications such as bleeding at a remote site proximal from the access site, swelling, fistula, pseudo-aneurysm and compartment syndrome. The location of the perforation might influence the severity of the complication and the impact on function. However, location was sparsely reported [93, 113, 184]. Again, functional outcome was not reported.

### Dissection

A dissection incidence of up to 0.7 % was shown by the pooled dissection data retrieved from 17 articles (Table 1; [8, 20, 22, 24, 26, 27, 37, 41, 50, 51, 73, 90, 101, 102, 186, 190, 192]). Anatomical variations, such as a higher bifurcation of the radial artery, are more prone to dissection [8]. Yet location of the dissection was sparsely reported [8, 22]. Only 1 article investigated functional outcome, which was not affected [22]. However, it is unclear how this was ascertained.

### Swelling

Five articles reported on swelling, with an incidence of up to 3.5 % (Table 1; [28, 34, 35, 80, 154]). It is often a benign symptom. Nevertheless, it can lead to permanent tissue damage, especially to the hand and arm. None of the articles described aetiology nor functional outcome at follow-up.

### Compartment syndrome

Compartment syndrome was described in 8 studies, up to an incidence of 0.14 % (Table 1; [8, 32, 37, 45, 51, 56, 173, 184]). Tizon-Marcos et al. retrospectively reviewed data of TR-PCP and described 2 cases both requiring fasciotomy [32]. One patient developed a pseudo-aneurysm which lead to compartment syndrome and retained permanent damage. The second patient showed complete neuromuscular recovery within 6 years. Aetiology was not ascertained. Also, two cases requiring emergency surgery after TR-PCP were described by Burzotta et al. They did not describe causality and functional outcome [37].

### Pseudo-aneurysm

A pooled incidence of 0.04 % for pseudo-aneurysm was reported in 52 articles ranging from requiring surgery to conservative treatment (Table 1; [20, 22, 24, 25, 27, 30, 31, 36–38, 40, 41, 43–45, 50, 51, 55–57, 60–62 ,69, 73, 96, 103, 112, 122, 124, 137–139, 144–146, 148, 155, 157, 166, 171–173, 180, 181, 183, 184, 188, 192]). Only 2 articles reported functional outcome in relationship to pseudo-aneurysm [22, 32]. Just one of them described permanent functional loss [22].

### Arteriovenous fistula

Arteriovenous fistula had a pooled incidence of up to 0.2 %, as was retrieved from 35 studies (Table 1; [25–27, 30, 31, 36–38, 40, 41, 43–45, 50, 51, 56, 57, 60–62, 95, 112, 113, 117, 136–138, 142, 144, 155, 166, 179, 184, 188, 191]). Fistula might lead to upper extremity dysfunction through swelling, which could lead to permanent disability depending on the severity and location. Nonetheless, neither the precise location of the fistula, nor the functional outcome were described.

### Infection/Inflammation

Seven articles mentioned infection and/or inflammation with a pooled incidence of up to 1.1 % (Table 1; [22, 28, 37, 73, 137, 171, 193]). Only 1 article described the absence of upper extremity dysfunction at follow-up [22]. Infection and/or inflammation is accompanied by swelling, pain and ‘functio laesa’. When untreated it can lead to permanent tissue damage in the complex anatomy of the hand and arm.

### Meta-regression analysis

The results of the random-effects model also demonstrated high statistical heterogeneity and significant high I^2^ values in nearly all outcomes. Therefore, a meta-regression analysis was conducted to reduce the heterogeneity associated with the incidence of access-site complications. Type of procedure was considered an important covariate in complication rate. In order to confirm this with our data a meta-regression analysis was conducted with procedure stratifications. Meta-regression analyses showed a significant positive correlation between TR-PCI and compartment syndrome (*p* = 0.0144) (Table 2). Moreover, the results for the correlation between early radial artery occlusion and type of procedure suggest a trend not beneficial for TR-CAG (*p* = 0.0605). All the other access-site complications showed no significant correlation with the type of procedure, which confirms that no difference should be made regarding the type of procedure, giving more value to studying all the procedures combined, thus TR-PCP. The funnel plot results were not considered robust against publication bias.

**Table 2:** Meta-regression analysis results for primary outcome with procedure as covariance. A test of moderators was run by R software programming language to find significant differences between access-site complications and type of procedure. TR-PCI shows more compartment syndrome (*p* = 0.01) and less early radial occlusion artery (*p* = 0.06) in comparison to TR-CAG

Complication	Qm	df	*P*-value
Upper extremity dysfunction	1.0262	2	0.5986
Upper extremity ischaemia	0.7596	2	0.6840
Pain	0.4701	2	0.7905
Radial artery spasm	3.2052	2	0.2014
Severe radial artery spasm	1.6600	2	0.4360
Early radial artery occlusion	5.0693	2	0.0605
Late radial artery occlusion	0.9621	3	0.8104
Minor access-site bleeding	1.6248	2	0.4438
Major access-site bleeding	2.9380	2	0.2302
Minor access-site haematoma	3.4631	2	0.1770
Major access-site haematoma	4.1858	2	0.1233
Perforation	0.8900	2	0.6408
Dissection	0.2113	2	0.8997
Swelling	1.7717	2	0.4124
Compartment syndrome	8.4838	2	0.0144^a^
Pseudo-aneurysm	1.0197	2	0.6006
Arteriovenous fistula	0.0378	2	0.9813
Infection/Inflammation	0.0380	1	0.8454

*TR-CAG* transradial catheterisation, *TR-PCI* transradial percutaneous coronary intervention, *Qm* Q-model, a measure of model fit.

^a^Significant effect of type of procedures on access site complication.

## Discussion

The transradial route is gaining popularity among intervention cardiologists mainly due to lower access-site bleeding and lower mortality than with the femoral approach [1–3]. With increased use of TR-PCP, upper extremity dysfunction may occur more frequently, stressing the need to optimise this technique.

The current review reports a mean incidence of known access-site complications of up to 9.6 %, which consist mainly of radial occlusion, radial spasm, swelling and haematoma (Table 1). Only 14 articles specifically mention upper extremity function after TR-PCP with a very low-pooled incidence of up to 0.8 %. Only 5 of these articles measured upper extremity dysfunction as an endpoint [20–24]. Two articles measured grip-strength [20, 21], yet only one as a primary endpoint [20]. This article described a small population, was retrospective in nature and used outdated equipment. In the other article it was used as a means to evaluate the Allen’s test and not for establishing functional loss [21]. Three articles evaluated functional loss at follow-up, but the evaluation strategy to determine upper extremity dysfunction was not or insufficiently described. The other 9 articles mentioned cases in which severe upper extremity dysfunction occurred. However, they did not measure it as a primary endpoint.

The current reported low incidence of access-site complications post TR-PCP is possibly an underestimation of the actual incidence of complications. Many of the articles were not primarily focused on access-site complications and there is a wide range of reported incidences. Additionally, there is structural underreporting of complications in the literature due to negative publication bias and inadequate follow-up [194]. Moreover, those centres that publish their data are often experienced in using the transradial approach. However, the complication rate is much higher among less experienced cardiologists, due to the learning curve [6]. This may lead to a discrepancy between the literature and day-to-day practice. Our relatively high pooled incidences for complications post TR-CAG supposedly also reflect an overestimation, while often less experienced cardiologists perform TR-CAG. In addition, these high incidences (namely in radial artery occlusion; 4.98 %), might be an effect of multiple catheter switches. Moreover, in the majority of the studies the definitions of the endpoints were not specified or unclear, which might give an ambiguous rendition of the complication rate. Our hypothesis for the significant positive correlation between compartment syndrome and TR-PCI is that it is caused by the higher dosage of anticoagulants used in TR-PCI, which causes major bleeding that can lead to compartment syndrome when not treated properly.

Access-site complications might have a direct relationship with upper extremity dysfunction. The mechanism in which previous complications affect upper extremity function is a complicated cascade of events. It includes, but is not limited to, intima damage, hypoxia, soft tissue injury, swelling and nerve damage [195, 196]. To illustrate this, it should be noted that the main nerves of the upper extremity initially are mixed—sensory and motoric [197]—and that they have their own blood supply. Damage to this vasa nervorum due to temporary occlusion after spasm can result in ischaemia and possibly irreversible nerve damage [34]. This affects the motor and sensory functions of the upper extremities, thus negatively affecting one or more of the physiological parameters of upper extremity function, with a potentially debilitating outcome (Fig. 2). The magnitude of which depends on anatomical variation and location of the complication (Fig. 1). Evaluation of upper extremity comprises several factors (Fig. 1). Taking these separate factors into account, the incidence of complications is expected to be even higher than 9.6 %. When considering the results, associations between complications and functional outcome are sparsely made in the current literature. However, aetiology and impact are key to understanding and predicting complications (Fig. 1).

The perspective and focus of a cardiologist differs from that of a hand surgeon for this type of complication. Consequently, a cardiologist could easily overlook upper extremity dysfunction. This is validated by our finding that only one article objectively measured upper extremity dysfunction as an endpoint after TR-CAG [20].

In addition, patients might be hesitant to complain about limb dysfunction after a major cardiac event and cardiologists might be untrained to investigate upper extremity function. When unnoticed, complications could be a precipitating factor in severe disability, with possibly significant socioeconomic consequences on patient and society [198]. To understand and prevent the effect of access-site complications on the function of the upper extremity, knowledge of the anatomy of the upper extremity and its numerous anatomical variations is essential (Fig. 1; [195]).

The radial artery is more susceptible to spasm, intimae damage and occlusion in some individuals, particularly females, the elderly and diabetics whereas the radial artery tends to be smaller in calibre [8, 196]. Such patients might benefit from prudence, perhaps being treated by expert radialists only. Prevention can also be achieved by slender percutaneous coronary procedures (PCP) with refinement of equipment [199]. Miniaturisation of guiding catheters, with diagnostic catheters as small as 3Fr, might be less traumatic and reduce the chance of occlusion and perforation [199]. The use of hydrophilic or sheathless catheters, with excellent torque control and large inner lumen while maintaining a small outer lumen, reduces friction and perhaps causes less abrasions to the intimae, [195] thus lowering the incidence of spasm and occlusion [200]. Furthermore, radial access closure devices give adequate pressure without obstructing the radial artery, thus lowering the occlusion rate [37]. Patients might benefit from early detection and treatment, since cases left untreated might lead to irreversible injury [32, 201, 202]. This emphasises the need to train cardiologists to signal upper extremity dysfunction, which could lead to early referral to a hand specialists.

Clearly, there are some limitations to this article. A review is dependent upon the information available in the literature and there are only 14 articles that mention upper extremity dysfunction after TR-PCP [20–33]. Access-site complications are frequently mentioned, but those articles report little about location, severity, causality, treatment and outcome. Therefore, several aspects of this article are based on speculation. To gain knowledge about the magnitude and impact of upper extremity dysfunction after TR-PCP further hypothesising research is needed.

## Conclusion

The transradial is favoured above femoral approach in percutaneous coronary procedures (PCP) due to a lower major bleeding rate and increased patient comfort. However, the pooled incidence of access-site complications is up to 9.6 %. Furthermore, the rate of upper extremity dysfunction is rarely investigated, hardly ever as a primary endpoint, and if investigated not thoroughly enough. Therefore, the incidence of upper extremity dysfunction is underestimated. Nonetheless, it could have significant socioeconomic impact. This study may provide a strong basis for a study design regarding upper extremity function after TR-PCP. Also, TR-PCP can be optimised by additional studies investigating the magnitude, prevention and best treatment of possible upper extremity dysfunction following the use of this technique.

## Supplementary references

A table with the remaining references, with in the first column the reference number corresponding with the references in the text. (PDF 167 KB)
