# Supplementary material for: Revealing the impact of local access-site complications and upper extremity dysfunction post transradial percutaneous coronary procedures
Source: Neth Heart J. 2015 Oct 5;23(11):514–24. doi: 10.1007/s12471-015-0747-9 (PMC4608927; doi:10.1007/s12471-015-0747-9)
Supplement: Supplementary file 1 — A table with the remaining references, with in the first column the reference number corresponding with the references in the text. (PDF 167 KB) [file 12471_2015_747_MOESM1_ESM.pdf]

Supplementary references

51. Leonardi RA, Townsend JC, Bonnema DD, et al. Comparison of percutaneous coronary intervention safety before and during the establishment of a transradial program at a teaching hospital. Am J Cardiol. 2012;109(8):1154–59.
52. Mann T, Cubeddu G, Bowen J, et al. Stenting in acute coronary syndromes: a comparison of radial versus femoral access sites. J Am Coll Cardiol. 1998;32(3):572–576.
53. Mann JT 3rd, Cubeddu MG, Schneider JE, et al. Right radial access for PTCA: a prospective study demonstrates reduced complications and hospital charges. J Invasive Cardiol. 1996;8(Suppl D)40D–4D.
54. Molinari G, Nicoletti I, De Benedictis M, et al. Safety and efficacy of the percutaneous radial artery approach for coronary angiography and angioplasty in the elderly. J Invasive Cardiol 2005;17(12):651–4.
55. Nagai S, Abe S, Sato T, et al. Ultrasonic assessment of vascular complications in coronary angiography and angioplasty after transradial approach. Am J Cardiol. 1999;83(2):180–6.
56. Nie B, Zhou YJ, Yang Q, et al. Safety and feasibility of repeated percutaneous transradial coronary intervention in the same route. Chin Med J (Engl). 2012;125(2):221–5.
57. Pancholy SB, Patel TM. Effect of duration of hemostatic compression on radial artery occlusion after transradial access. Catheter Cardiovasc Interv. 2012;79(1):78–81.
58. Philippe F, Larrazet F, Meziane T, et al. Comparison of transradial vs. transfemoral approach in the treatment of acute myocardial infarction with primary angioplasty and abciximab. Catheter Cardiovasc Interv. 2004;61(6):67–73.
59. Ranjan A, Patel TM, Shah SC, et al. Transradial primary angioplasty and stenting in Indian patients with acute myocardial infarction: acute results and 6-month follow-up. Indian Heart J. 2005;57(6):681–7.
60. Sciahbasi A, Mancone M, Cortese B, et al. Transradial percutaneous coronary interventions using sheathless guiding catheters: a multicenter registry. J Interv Cardiol. 2011;24(5):407–12.
61. Sciahbasi A, Romagnoli E, Burzotta F, et al. Transradial approach (left vs right) and procedural times during percutaneous coronary procedures: TALENT study. Am Heart J. 2011;161(1):172–9.
62. Slagboom T, Kiemeneij F, Laarman GJ, et al. Outpatient coronary angioplasty: feasible and safe. Catheter Cardiovasc Interv. 2005;64(4):421–7.
63. Stella PR, Kiemeneij F, Laarman GJ, et al. Incidence and outcome of radial artery occlusion following transradial artery coronary angioplasty. Cathet Cardiovasc Diagn. 1997;40(2):156–8.
64. Valsecchi O, Musumeci G, Vassileva A, et al. Safety, feasibility and efficacy of transradial primary angioplasty in patients with acute myocardial infarction. Ital Heart J. 2003;4(5):329–34.
65. Yoo BS, Lee SH, Ko JY, et al. Procedural outcomes of repeated transradial coronary procedure. Catheter Cardiovasc Interv. 2003;58(3):301–4.
66. Yoo BS, Yoon J, Ko JY, et al. Anatomical consideration of the radial artery for transradial coronary procedures: arterial diameter, branching anomaly and vessel tortuosity. Int J Cardiol. 2005;101(3):421–7.
67. Buturak A, Gorgulu S, Norgaz T, et al. The long term incidence and predictors of radial artery occlusion following a transradial coronary procedure. Cardiol J. 2014;21(4):350–6.
68. Chugh SK. Transradial access: right or left? Indian Heart J. 2008;60(1 Suppl A):A27–35.
69. Dery JP, Simard S, Barbeau GR. Reduction of discomfort at sheath removal during transradial coronary procedures with the use of a hydrophilic-coated sheath. Catheter Cardiovasc Interv. 2001;54(3):289–94.
70. Grinfeld L, Berrocal D, Rojas Matas C, et al. What is the most effective vascular approach for a diagnostic cardiac catheterization? A randomized trial using the femoral, brachial or radial approaches. J Am Coll Cardiol. 1996;27(2s1):17–17.
71. Jia DA, Zhou YJ, Shi DM, et al. Incidence and predictors of radial artery spasm during transradial coronary angiography and intervention. Chin Med J (Engl). 2010;123(7):843–7.
72. Kindel M, Rüppel R. Hydrophilic-coated sheaths increase the success rate of transradial coronary procedures and reduce patient discomfort but do not reduce the occlusion rate: randomized single-blind comparison of coated vs. non-coated sheaths. Clin Res Cardiol. 2008;97(9):609–14.
73. Rathore S, Stables RH, Pauriah M, et al. Impact of length and hydrophilic coating of the introducer sheath on radial artery spasm during transradial coronary intervention: a randomized study. JACC Cardiovasc Interv. 2010;3(5):475–83.
74. Sansanayudh N, Champasri K, Piamsomboon C, et al. The efficacy and safety of transradial approach in comparison with transfemoral approach for coronary angiography and ad hoc coronary angioplasty in Thailand. J Med Assoc Thai. 2010;93(Suppl 6):S145–51.
75. Kim SH, Kim EJ, Cheon WS, et al. Comparative study of nicorandil and a spasmolytic cocktail in preventing radial artery spasm during transradial coronary angiography. Int J Cardiol. 2007;120(3):325–30.
76. Aminian A, Dolatabadi D, Lefebvre P, et al. Initial experience with the glidesheath slender for transradial coronary angiography and intervention: A feasibility study with prospective radial ultrasound follow-up. Catheter Cardiovasc Interv. 2014;84(3):436–42.
77. Kiemeneij F, Vajifdar BU, Eccleshall SC, et al. Evaluation of a spasmolytic cocktail to prevent radial artery spasm during coronary procedures. Catheter Cardiovasc Interv. 2003;58(3):281–4.
78. Biondi-Zoccai GG, Moretti C, Omedè P, et al. Intra-arterial lidocaine versus saline to reduce peri-procedural discomfort in patients undergoing percutaneous trans-radial or trans-ulnar coronary procedures. Acta Cardiol. 2011;66(1):9–14.
79. Caussin C, Gharbi M, Durier C, et al. Reduction in spasm with a long hydrophilic transradial sheath. Catheter Cardiovasc Interv. 2010;76(5):668–72.
80. Gwon HC, Doh JH, Choi JH, et al. A 5Fr catheter approach reduces patient discomfort during transradial coronary intervention compared with a 6Fr approach: a prospective randomized study. J Interv Cardiol. 2006;19(2):141–7.
81. Coppola J, Patel T, Kwan TI. Nitroglycerin, nitroprusside, or both, in preventing radial artery spasm during transradial artery catheterization. J Invasive Cardiol. 2006;18(4):155–8.
82. Deftereos S, Giannopoulos G, Kossyvakis C, et al. Radial artery flow-mediated dilation predicts arterial spasm during transradial coronary interventions. Catheter Cardiovasc Interv. 2011;77(5):649–54.
83. Deftereos S, Giannopoulos G, Raisakis K, et al. Moderate procedural sedation and opioid analgesia during transradial coronary interventions to prevent spasm: a prospective randomized study. JACC Cardiovasc Interv. 2013;6(3):267–73.
84. Goldsmit A, Kiemeneij F, Gilchrist IC, et al. Radial artery spasm associated with transradial cardiovascular procedures: results from the RAS registry. Catheter Cardiovasc Interv. 2014;83(1):E32–6.
85. Gorgulu S, Norgaz T, Karaahmet T, et al. Incidence and predictors of radial artery spasm at the beginning of a transradial coronary procedure. J Interv Cardiol. 2013;26(2):208–13.
86. Hahalis G, Tsigkas G, Xanthopoulou I, et al. Transulnar compared with transradial artery approach as a default strategy for coronary procedures: a randomized trial. The Transulnar or Transradial Instead of Coronary Transfemoral Angiographies Study (the AURA of ARTEMIS Study). Circ Cardiovasc Interv. 2013;6(3):252–61.

87. Kiemeneij F, Fraser D, Slagboom T, et al. Hydrophilic coating aids radial sheath withdrawal and reduces patient discomfort following transradial coronary intervention: a randomized double-blind comparison of coated and uncoated sheaths. Catheter Cardiovasc Interv. 2003;59(2):161–4.
88. Koga S, Ikeda S, Futagawa K, et al. The use of a hydrophilic-coated catheter during transradial cardiac catheterization is associated with a low incidence of radial artery spasm. Int J Cardiol. 2004;96(2):255–8.
89. Mamas M, D'Souza S, Hendry C, et al. Use of the sheathless guide catheter during routine transradial percutaneous coronary intervention: a feasibility study. Catheter Cardiovasc Interv. 2010;75(4):596–602.
90. Norgaz T, Gorgulu S, Dagdelen S. A randomized study comparing the effectiveness of right and left radial approach for coronary angiography. Catheter Cardiovasc Interv. 2012;80(2):260–4.
91. Ouadhour A, Sideris G, Smida W, et al. Usefulness of subcutaneous nitrate for radial access. Catheter Cardiovasc Interv. 2008;72(3):343–6.
92. Park KH, Park DW, Kim MK, et al. Effects of sheath injury and trimetazidine on endothelial dysfunction of radial artery after transradial catheterization. J Interv Cardiol. 2012;25(4):411–7.
93. Ruiz-Salmerón RJ, Mora R, Masotti M, et al. Assessment of the efficacy of phentolamine to prevent radial artery spasm during cardiac catheterization procedures: a randomized study comparing phentolamine vs. verapamil. Catheter Cardiovasc Interv. 2005;66(2):192–8.
94. Saito S, Tanaka S, Hiroe Y, et al. Usefulness of hydrophilic coating on arterial sheath introducer in transradial coronary intervention. Catheter Cardiovasc Interv. 2002;56(3):328–32.
95. Tse TS, Lam KK, Tsui KL, et al. Feasibility of transradial coronary angiography and angioplasty in Chinese patients. Hong Kong Med J. 2006;12(2):108–14.
96. Tuncez A, Kaya Z, Aras D, et al. Incidence and predictors of radial artery occlusion associated transradial catheterization. Int J Med Sci. 2013;10(12):1715–9.
97. Varenne O, Jégou A, Cohen R, et al. Prevention of arterial spasm during percutaneous coronary interventions through radial artery: the SPASM study. Catheter Cardiovasc Interv. 2006;68(2):231–5.
98. Waggoner T, White R, Wassmer PV, et al. Radial artery spasm prevention (RASP study): a retrospective analysis of oral pharmacologic prophylaxis for prevention of radial artery spasm during transradial access for cardiac catheterization. Cath lab Digest. 2014;22(7):1–26
99. Abdelaal E, Brousseau-Provencher C, Montminy S, et al. Risk score, causes, and clinical impact of failure of transradial approach for percutaneous coronary interventions. JACC Cardiovasc Interv. 2013;6(11):1129–37.
100. Dahm JB, Vogelgesang D, Hummel A, et al. A randomized trial of 5 vs. 6 French transradial percutaneous coronary interventions. Catheter Cardiovasc Interv. 2002;57(2):172–6.
101. Dehghani P, Mohammad A, Bajaj R, et al. Mechanism and predictors of failed transradial approach for percutaneous coronary interventions. JACC Cardiovasc Interv. 2009;2(11):1057–64.
102. Gellen B, Lesault PF, Canouï-Poitrine F, et al. Feasibility limits of transradial primary percutaneous coronary intervention in acute myocardial infarction in the real life (TRAP-AMI). Int J Cardiol. 2013;168(2):1056–61.
103. Hou L, Wei YD, Li WM, et al. Comparative study on transradial versus transfemoral approach for primary percutaneous coronary intervention in Chinese patients with acute myocardial infarction. Saudi Med J. 2010;31(2):158–62.
104. Kwan TW, Cherukuri S, Huang Y, et al. Feasibility and safety of 7F sheathless guiding catheter during transradial coronary intervention. Catheter Cardiovasc Interv. 2012;80(2):274–80.
105. Li WM, Li Y, Zhao JY, et al. Safety and feasibility of emergent percutaneous coronary intervention with the transradial access in patients with acute myocardial infarction. Chin Med J (Engl). 2007;120(7):598–600.
106. Louvard Y, Ludwig J, Lefèvre T, et al. Transradial approach for coronary angioplasty in the setting of acute myocardial infarction: a dual-center registry. Catheter Cardiovasc Interv. 2002;55(2):206–11.
107. Rathore S, Roberts E, Hakeem AR, et al. The feasibility of percutaneous transradial coronary intervention for saphenous vein graft lesions and comparison with transfemoral route. J Interv Cardiol. 2009;22(4):336–40.
108. Rathore S, Hakeem A, Pauriah M, et al. A comparison of the transradial and the transfemoral approach in chronic total occlusion percutaneous coronary intervention. Catheter Cardiovasc Interv. 2009;73(7):883–7.
109. Vink MA, Amoroso G, Dirksen MT, et al. Routine use of the transradial approach in primary percutaneous coronary intervention: procedural aspects and outcomes in 2209 patients treated in a single high-volume centre. Heart. 2011;97(23):1938–42.
110. Yiğit F, Sezgin AT, Erol T, et al. An experience on radial versus femoral approach for diagnostic coronary angiography in Turkey. Anadolu Kardiyol Derg. 2006;6(3):229–34.
111. Ziakas A, Klinke P, Mildenberger R, et al. A comparison of the radial and the femoral approach in vein graft PCI. A retrospective study. Int J Cardiovasc Intervent. 2005;7(2):93–6.
112. Ziakas AG, Koskinas KC, Gavrilidis S, et al. Radial versus femoral access for orally anticoagulated patients. Catheter Cardiovasc Interv. 2010;76(4):493–9.
113. Ziakas A, Klinke P, Mildenberger R, et al. Comparison of the radial and the femoral approaches in percutaneous coronary intervention for acute myocardial infarction. Am J Cardiol. 2003;91(5):598–600.
114. Chen CW, Lin CL, Lin TK, et al. A simple and effective regimen for prevention of radial artery spasm during coronary catheterization. Cardiology. 2006;105(1):43–7.
115. Bertrand B, Sene Y, Huygue O, et al. Doppler ultrasound imaging of the radial artery after catheterization. Ann Cardiol Angeiol 2003;52(3):135–8.
116. Cheng KY, Chair SY, Choi KC. Access site complications and puncture site pain following transradial coronary procedures: a correlational study. Int J Nurs Stud. 2013;50(10):1304–13.
117. Chodór P, Krupa H, Kurek T, et al. RADIal versus femoral approach for percutaneous coronary interventions in patients with Acute Myocardial Infarction (RADIAMI): a prospective, randomized, single-center clinical trial. Cardiol J. 2009;16(4):332–40.
118. Cooper CJ, El-Shiekh RA, Cohen DJ, et al. Effect of transradial access on quality of life and cost of cardiac catheterization: a randomized comparison. Am Heart J. 1999;138(3 Pt 1):430–6.
119. Cubero JM, Lombardo J, Pedrosa C, et al. Radial compression guided by mean artery pressure versus standard compression with a pneumatic device (RACOMAP). Catheter Cardiovasc Interv. 2009;73(4):467–72.
120. Gobeil F, Brück M, Louvard Y, et al. Comparison of 5 French versus 6 French guiding catheters for transradial coronary intervention: a prospective, randomized study. J Invasive Cardiol. 2004;16(7):353–5.
121. Honda T, Fujimoto K, Miyao Y, et al. Access site-related complications after transradial catheterization can be reduced with smaller sheath size and statins. Cardiovasc Interv Ther 2012;27(3):174–80.

122. Kelbaek H, Vogt K, Nielsen T, et al. Percutaneous transradial coronary angiography and angioplasty in patients with occlusive atherosclerotic iliofemoral disease. Scand Cardiovasc J. 2000;34(1):84–6.
123. Louvard Y, Lefèvre T, Allain A, et al. Coronary angiography through the radial or the femoral approach: The CARAFE study. Catheter Cardiovasc Interv. 2001;52(2):181–7.
124. Louvard Y, Benamer H, Garot P, et al. Comparison of transradial and transfemoral approaches for coronary angiography and angioplasty in octogenarians (the OCTOPLUS study). Am J Cardiol. 2004;94(9):1177–80.
125. Mann T, Cowper PA, Peterson ED, et al. Transradial coronary stenting: comparison with femoral access closed with an arterial suture device. Catheter Cardiovasc Interv. 2000;49(2):150–6.
126. Pancholy SB. Comparison of the effect of intra-arterial versus intravenous heparin on radial artery occlusion after transradial catheterization. Am J Cardiol. 2009;104(8):1083–5.
127. Pancholy S, Coppola J, Patel T. et al. Prevention of radial artery occlusion-patent hemostasis evaluation trial (PROPHET study): a randomized comparison of traditional versus patency documented hemostasis after transradial catheterization. Catheter Cardiovasc Interv. 2008;72(3):335–40.
128. Pancholy SB, Bertrand OF, Patel T. Comparison of a priori versus provisional heparin therapy on radial artery occlusion after transradial coronary angiography and patent hemostasis (from the PHARAOH Study). Am J Cardiol. 2012;110(2):173–6.
129. Pancholy SB, Sanghvi KA, Patel TM. Radial artery access technique evaluation trial: randomized comparison of Seldinger versus modified Seldinger technique for arterial access for transradial catheterization. Catheter Cardiovasc Interv. 2012;80(2):288–91.
130. Pancholy SB. Impact of two different hemostatic devices on radial artery outcomes after transradial catheterization. J Invasive Cardiol. 2009;21(3):101–4.
131. Politi L, Aprile A, Paganelli C, et al. Randomized clinical trial on short-time compression with Kaolin-filled pad: a new strategy to avoid early bleeding and subacute radial artery occlusion after percutaneous coronary intervention. J Interv Cardiol. 2011;24(1):65–72.
132. Rathore S, Stables RH, Pauriah M, et al. A randomized comparison of TR band and radistop hemostatic compression devices after transradial coronary intervention. Catheter Cardiovasc Interv. 2010;76(5):660–7.
133. Sanmartin M, Goicolea J, Ocaranza R, et al. Vasoreactivity of the radial artery after transradial catheterization. J Invasive Cardiol. 2004;16(11):635–8.
134. Santas E, Bodí V, Sanchis J, et al. The left radial approach in daily practice. A randomized study comparing femoral and right and left radial approaches. Rev Esp Cardiol. 2009;62(5):482–90.
135. Shen H, Zhou YJ, Liu YY, et al. Assessment of early radial injury after transradial coronary intervention by high-resolution ultrasound biomicroscopy: innovative technology application. Chin Med J (Engl). 2012;125(19):3388–92.
136. Takeshita S, Asano H, Hata T, Hibi K, et al. Comparison of frequency of radial artery occlusion after 4Fr versus 6Fr transradial coronary intervention (from the Novel Angioplasty USIng Coronary Accessor Trial). Am J Cardiol. 2014;113(12):1986–9.
137. Takeshita S, Shiono T, Takagi A, et al. Percutaneous coronary intervention using a novel 4-French coronary accessor. Catheter Cardiovasc Interv. 2008;72(2):222–7.
138. Uhlemann M, Möbius-Winkler S, Mende M, et al. The Leipzig prospective vascular ultrasound registry in radial artery catheterization: impact of sheath size on vascular complications. JACC Cardiovasc Interv. 2012;5(1):36–43.
139. Wang YB, Fu XH, Wang XC, et al. Randomized comparison of radial versus femoral approach for patients with STEMI undergoing early PCI following intravenous thrombolysis. J Invasive Cardiol. 2012;24(8):412–6.
140. Yan Z, Zhou Y, Zhao Y, et al. Impact of transradial coronary procedures on radial artery. Angiology. 2010;61(1):8–13.
141. Sanmartin M, Gomez M, Rumoroso JR, et al. Interruption of blood flow during compression and radial artery occlusion after transradial catheterization. Catheter Cardiovasc Interv. 2007;70(2):185–9.
142. Cantor WJ, Puley G, Natarajan MK, et al. Radial versus femoral access for emergent percutaneous coronary intervention with adjunct glycoprotein IIb/IIIa inhibition in acute myocardial infarction–the RADIAL-AMI pilot randomized trial. Am Heart J. 2005;150(3):543–9.
143. Plante S, Cantor WJ, Goldman L, et al. Comparison of bivalirudin versus heparin on radial artery occlusion after transradial catheterization. Catheter Cardiovasc Interv. 2010;76(5):654–8.
144. Achenbach S, Ropers D, Kallert L, et al. Transradial versus transfemoral approach for coronary angiography and intervention in patients above 75 years of age. Catheter Cardiovasc Interv. 2008;72(5):629–35.
145. Hibbert B, Simard T, Wilson KR, et al. Transradial versus transfemoral artery approach for coronary angiography and percutaneous coronary intervention in the extremely obese. JACC Cardiovasc Interv. 2012;5(8):819–26.
146. Hu F, Yang Y, Qiao S, et al. Comparison between radial and femoral approach for percutaneous coronary intervention in patients aged 80 years or older. J Interv Cardiol. 2012;25(5):513–7.
147. Jabara R, Gadesam R, Pendyala L, et al. Ambulatory discharge after transradial coronary intervention: Preliminary US single-center experience (Same-day TransRadial Intervention and Discharge Evaluation, the STRIDE Study). Am Heart J. 2008;156(6):1141–6.
148. Koutouzis M, Matejka G, Olivecrona G, et al. Radial vs. femoral approach for primary percutaneous coronary intervention in octogenarians. Cardiovasc Revasc Med. 2010;11(2):79–83.
149. Romagnoli E, De Vita M, Burzotta F, et al. Radial versus femoral approach comparison in percutaneous coronary intervention with intraaortic balloon pump support: the RADIAL PUMP UP registry. Am Heart J. 2013;166(6):1019–26.
150. Small A, Klinke P, Della Siega A, et al. Day procedure intervention is safe and complication free in higher risk patients undergoing transradial angioplasty and stenting. The discharge study. Catheter Cardiovasc Interv. 2007;70(7):907–12.
151. Bernat I, Abdelaal E, Plourde G, et al. Early and late outcomes after primary percutaneous coronary intervention by radial or femoral approach in patients presenting in acute ST-elevation myocardial infarction and cardiogenic shock. Am Heart J. 2013;165(3):338–43.
152. Bertrand OF, Rodés-Cabau J, Rinfret S, et al. Impact of final activated clotting time after transradial coronary stenting with maximal antiplatelet therapy. Am J Cardiol. 2009;104(9):1235–40.
153. Bertrand OF, Bagur R, Costerousse O, et al. Transradial vs femoral percutaneous coronary intervention for left main disease in octogenarians. Indian Heart J. 2010;62(3):234–7.
154. Chaumeil A, Beygui F, Collet JP, et al. Feasibility of outpatient coronary angiography with „ad hoc“ angioplasty. Arch Cardiovasc Dis. 2008;101(6):383–90.
155. De Carlo M, Borelli G, Gistri R, et al. Effectiveness of the transradial approach to reduce bleedings in patients undergoing urgent coronary angioplasty with GPIIb/IIIa inhibitors for acute coronary syndromes. Catheter Cardiovasc Interv. 2009;74(3):408–15.

156. Hamon M, Rasmussen LH, Manoukian SV, et al. Choice of arterial access site and outcomes in patients with acute coronary syndromes managed with an early invasive strategy: the ACUITY trial. EuroIntervention. 2009;5(1):115–20.
157. Hetherington SL, Adam Z, Morley R, et al. Primary percutaneous coronary intervention for acute ST-segment elevation myocardial infarction: changing patterns of vascular access, radial versus femoral artery. Heart. 2009;95(19):1612–8.
158. Jaffe R, Hong T, Sharieff W, et al. Comparison of radial versus femoral approach for percutaneous coronary interventions in octogenarians. Catheter Cardiovasc Interv. 2007;69(6):815–20.
159. Kassam S, Cantor WJ, Patel D, et al. Radial versus femoral access for rescue percutaneous coronary intervention with adjuvant glycoprotein IIb/IIIa inhibitor use. Can J Cardiol. 2004;20(14):1439–42.
160. Maynard C, Bradley SM, Bryson CL. The practice of transradial percutaneous coronary intervention in the Washington State Clinical Outcomes Assessment Program. Am Heart J. 2013;165(3):332–7.
161. Natsuaki M, Morimoto T, Furukawa Y, et al. Comparison of 3-year clinical outcomes after transradial versus transfemoral percutaneous coronary intervention. Cardiovasc Interv Ther. 2012;27(2):84–92.
162. Numasawa Y, Kohsaka S, Miyata H, et al. Safety of transradial approach for percutaneous coronary intervention in relation to body mass index: a report from a Japanese multicenter registry. Cardiovasc Interv Ther. 2013;28(2):148–56.
163. Ochiai M, Isshiki T, Toyoizumi H, et al. Efficacy of transradial primary stenting in patients with acute myocardial infarction. Am J Cardiol. 1999;83(6):966–8.
164. Romagnoli E, Biondi-Zoccai G, Sciahbasi A, et al. Radial versus femoral randomized investigation in ST-segment elevation acute coronary syndrome: the RIFLE-STEACS (Radial Versus Femoral Randomized Investigation in ST-Elevation Acute Coronary Syndrome) study. J Am Coll Cardiol. 2012;60(24):2481–9.
165. Secco GG, Marinucci L, Uguccioni L. Transradial versus transfemoral approach for primary percutaneous coronary interventions in elderly patients. J Invasive Cardiol. 2013;25(5):254–6.
166. Yip HK, Chung SY, Chai HT, et al. Safety and efficacy of transradial vs transfemoral arterial primary coronary angioplasty for acute myocardial infarction: single-center experience. Circ J. 2009;73(11):2050–5.
167. Bertrand OF, De Larochellière R, Rodés-Cabau J, et al. Early Discharge After Transradial Stenting of Coronary Arteries Study Investigators. A randomized study comparing same-day home discharge and abciximab bolus only to overnight hospitalization and abciximab bolus and infusion after transradial coronary stent implantation. Circulation. 2006;114(24):2636–43.
168. Brasselet C, Tassan S, Nazeyrollas P, et al. Randomised comparison of femoral versus radial approach for percutaneous coronary intervention using abciximab in acute myocardial infarction: results of the FARMI trial. Heart. 2007;93(12):1556–61.
169. Cafri C, Zahger D, Merkin M, et al. Efficacy of the radial approach for the performance of primary PCI for STEMI. J Invasive Cardiol. 2013;25(3):150–3.
170. Ibebuogu UN, Cercek B, Makkar R, et al. Comparison between transradial and transfemoral percutaneous coronary intervention in acute ST-elevation myocardial infarction. Am J Cardiol. 2012;110(9):1262–5.
171. Pancholy S, Patel T, Sanghvi K, et al. Comparison of door-to-balloon times for primary PCI using transradial versus transfemoral approach. Catheter Cardiovasc Interv. 2010;75(7):991–5.
172. Reddy BK, Brewster PS, Walsh T, et al. Randomized comparison of rapid ambulation using radial, 4 French femoral access, or femoral access with AngioSeal closure. Catheter Cardiovasc Interv. 2004;62(2):143–9.
173. Ruzsa Z, Ungi I, Horváth T, et al. Five-year experience with transradial coronary angioplasty in ST-segment-elevation myocardial infarction. Cardiovasc Revasc Med. 2009;10(2):73–9.
174. Tizón-Marcos H, Bertrand OF, Rodés-Cabau J, et al. Impact of female gender and transradial coronary stenting with maximal antiplatelet therapy on bleeding and ischemic outcomes. Am Heart J. 2009;157(4):740–5.
175. Pristipino C, Pelliccia F, Granatelli A, et al. Comparison of access-related bleeding complications in women versus men undergoing percutaneous coronary catheterization using the radial versus femoral artery. Am J Cardiol. 2007;99(9):1216-21.
176. Bernat I, Horak D, Stasek J, et al. ST-segment elevation myocardial infarction treated by radial or femoral approach in a multicenter randomized clinical trial: the STEMI-RADIAL trial. J Am Coll Cardiol. 2014;63(10):964–72.
177. Burzotta F, Trani C, Tommasino A, et al. Impact of operator experience and wiring technique on procedural efficacy of trans-radial percutaneous chronic total occlusion recanalization performed by dedicated radialists. Cardiol J 2013;20(5):560–7.
178. Chodór P, Kurek T, Kowalczuk A, et al. Radial vs femoral approach with StarClose clip placement for primary percutaneous coronary intervention in patients with ST-elevation myocardial infarction. RADIAMI II: a prospective, randomised, single centre trial. Kardiol Pol. 2011;69(8):763–71.
179. Généreux P, Mehran R, Palmerini T, et al. Radial access in patients with ST-segment elevation myocardial infarction undergoing primary angioplasty in acute myocardial infarction: the HORIZONS-AMI trial. EuroIntervention. 2011;7(8):905–16.
180. Gilchrist IC, Moyer CD, Gascho JA. Transradial right and left heart catheterizations: a comparison to traditional femoral approach. Catheter Cardiovasc Interv. 2006;67(4):585–8.
181. Hamon M, Mehta S, Steg PG, et al. Impact of transradial and transfemoral coronary interventions on bleeding and net adverse clinical events in acute coronary syndromes. EuroIntervention. 2011;7(1):91–7.
182. Le Corvoisier P, Gellen B, Lesault PF, et al. Ambulatory transradial percutaneous coronary intervention: a safe, effective, and cost-saving strategy. Catheter Cardiovasc Interv. 2013;81(1):15–23.
183. Qin X, Xiong W, Wang L, et al. Clinical investigation of transradial access for emergent percutaneous coronary intervention in patients with acute myocardial infarction. Clin Interv Aging. 2013;81(1):15–23.
184. Sanmartín M, Cuevas D, Goicolea J, et al. Vascular complications associated with radial artery access for cardiac catheterization. Rev Esp Cardiol. 2004;57(6):581–4.
185. Shaikh AH, Hanif B, Pathan A, et al. Transradial primary percutaneous coronary intervention—experience from a tertiary care cardiac centre. J Pak Med Assoc. 2013;63(6):731–4.
186. Shin JS, Tahk SJ, Yang HM, et al. Impact of female gender on bleeding complications after transradial coronary intervention (from the Korean Transradial Coronary Intervention registry). Am J Cardiol. 2014;113(12):2002–6.
187. Siudak Z, Zawislak B, Dziewierz A, et al. Transradial approach in patients with ST-elevation myocardial infarction treated with abciximab results in fewer bleeding complications: data from EUROTRANSFER registry. Coron Artery Dis. 2010;21(5):292–7.
188. Weaver AN, Henderson RA, Gilchrist IC, et al. Arterial access and door-to-balloon times for primary percutaneous coronary intervention in patients presenting with acute ST-elevation myocardial infarction. Catheter Cardiovasc Interv. 2010;75(5):695–9.
189. Ziakas A, Klinke P, Mildenberger R, et al. Comparison of the radial and femoral approaches in left main PCI: a retrospective study. J Invasive Cardiol. 2004;16(3):129–32.

190. Michael TT, Alomar M, Papayannis A, et al. A Randomized Comparison of the Transradial and Transfemoral Approaches for Coronary Artery Bypass Graft Angiography and Intervention (the RADIAL-CABG Trial). 2013. JACC Cardiovasc Interv. 2013;6(11):1138–44.
191. Calviño-Santos RA, Vázquez-Rodríguez JM, Salgado-Fernández J, et al. Management of iatrogenic radial artery perforation. Catheter Cardiovasc Interv. 2004;61(1):74–8.
192. Nadarasa K, Robertson MC, Wong CK, et al. Rapid cycle change to predominantly radial access coronary angiography and percutaneous coronary intervention: effect on vascular access site complications. Catheter Cardiovasc Interv. 2012;79(4):589–94.
193. Kozak M, Adams DR, Ioffreda MD, et al. Sterile inflammation associated with transradial catheterization and hydrophilic sheaths. Catheter Cardiovasc Interv. 2003;59(2):207–13.
194. Kicinski M. How does under-reporting of negative and inconclusive results affect the false-positive rate in meta-analysis? A simulation study. BMJ Open. 2014;4(8):e004831.
195. Yonetsu T, Kakuta T, Lee T, et al. Assessment of acute injuries and chronic intimal thickening of the radial artery after transradial coronary intervention by optical coherence tomography. Eur Heart J. 2010;31(13):1608–15.
196. Burstein JM, Gidrewicz D, Hutchison SJ, et al. Impact of radial artery cannulation for coronary angiography and angioplasty on radial artery function. Am J Cardiol. 2007;99(4):457–9.
197. Von Lanz T, Wachsmuth W. Äste des plexus brachialis. ln: Von Lanz T, Wachsmuth W, editors. Praktische Anatomie. Arm: Ein Lehr- und Hilfsbuch der anatomischen Grundlagen ärztlichen Handelns. Berlin: Springer-Verlag; 2004
198. Jaquet JB, Jagt I van der, Kuypers PD, et al. Spaghetti wrist trauma: functional recovery, return to work, and psychological effects. Plast Reconstr Surg. 2005;115(6):1609–17.
199. Kiemeneij F, Yoshimachi F, Matsukage T, et al. Focus on maximal miniaturisation of transradial coronary access materials and techniques by the Slender Club Japan and Europe: an overview and classification. EuroIntervention. 2015;10(10):1178–86.
200. Mamas MA, Fraser DG, Ratib K, et al. Minimising radial injury: prevention is better than cure. EuroIntervention. 2014;10(7):824–32.
201. Rappard JRM van, Overgoo MLE, Fritschy WM, et al. Volkmannse contractuur na acuut compartimentsyndroom van de bovenste extremiteit. Nederlandse Tijdschrift voor Plastische Chirurgie. 2013;4(4):137–9
202. Volkmann R. Die ischaemischen muskellahmungen und kontrakturen. Zentralbl Chir. 1881;8:801–3.
